# mTOR Inhibitors Induce Erythropoietin Resistance in Renal Transplant Recipients

**DOI:** 10.3389/fmed.2022.722058

**Published:** 2022-02-22

**Authors:** Reece Jefferies, Harish Puttagunta, Anoushka Krishnan, Ashley Irish, Ramyasuda Swaminathan, John K. Olynyk

**Affiliations:** ^1^Department of Nephrology and Renal Transplant, Fiona Stanley Hospital, Perth, WA, Australia; ^2^Department of Gastroenterology, Fiona Stanley Hospital, Perth, WA, Australia; ^3^School of Medical and Health Sciences, Edith Cowan University, Joondalup, WA, Australia

**Keywords:** renal transplant, mTOR inhibitor, anemia, iron deficiency, hepcidin, erythropoietin, erythropoietin resistance

## Abstract

**Aim:**

To elucidate the role of mTOR inhibitors on iron, hepcidin and erythropoietin-mediated regulation of hemopoiesis in stable renal transplant recipients (RTR).

**Background:**

Impaired hemopoiesis is common following renal transplantation managed using mTOR inhibitors. The mechanisms responsible are uncertain but include direct effects on iron, hepcidin or erythropoietin-mediated hemopoiesis.

**Methods:**

We conducted a single center prospective case-control study of 26 adult RTR with stable allograft function. RTR received stable mTOR dosing (cases, 11/26 [42%]) or stable tacrolimus dosing (controls, 15/26 [58%]). Baseline demographics, full blood count, renal function, iron studies, hepcidin-25, Interleukin-6 (IL-6) and erythropoietin (EPO) levels were determined.

**Results:**

There were no differences in age, gender or allograft function. Mean daily sirolimus dose for cases was 1.72 mg, with mean trough level of 8.46 ng/mL. Mean daily tacrolimus dose for controls was 4.3 mg, with mean trough level of 5.8 ng/mL. There were no differences in mean hemoglobin (143 vs. 147 g/L; *p* = 0.59), MCV (88 vs. 90 fL; *p* = 0.35), serum ferritin (150 vs. 85.7 μg/L; *p* = 0.06), transferrin saturation (26 vs. 23.3%; *p* = 0.46), IL-6 (11 vs. 7.02 pg/ml; *p* = 0.14) or hepcidin-25 (3.62 vs. 3.26 nM; *p* = 0.76) between the groups. EPO levels were significantly higher in the group receiving mTOR therapy (16.8 vs. 8.49 IU/L; *p* = 0.028). On logistic regression analysis EPO level was the only variable that had a significant impact providing an odds ratio of 0.84 (95%CI 0.66–0.98). The area under the receiver operator characteristic curve (ROC) for the analysis was 0.77 (95%CI 0.54–0.94) with *p* = 0.04.

**Conclusion:** Higher levels of EPO in the absence of deranged iron biochemistry or hepcidin-25 levels suggest that EPO resistance rather than impaired iron metabolism may contribute to the impaired hemopoiesis previously demonstrated in RTR on mTOR therapy.

## Introduction

Anemia occurs in 13–70% of individuals (1–3) following renal transplantation. Potential contributors to anemia include impaired graft function, drugs, inflammation, infection, iron deficiency and rejection. Mammalian target of rapamycin (mTOR) inhibitors have been shown to impair haemopoiesis and cause microcytosis (4, 5). The anti-proliferative effect of this class of drugs is evidenced by the leukopenia and thrombocytopenia associated with their use (6). The mechanism by which mTOR inhibitors exert their effects on red blood cells is uncertain. Thanaut et al. suggested that sirolimus induced anemia (SIA) is most likely due to an inflammatory state (7), plausible on the basis of the known effects of inflammation on iron bioavailability and hepcidin production. Hepcidin is a 25 amino acid peptide, produced by hepatocytes, and which acts as a negative regulator of iron release from cells by binding to ferroportin, resulting in its internalization and degradation (8). Hepcidin gene inactivation results in iron overload (9) whilst over-expression results in severe iron deficiency anemia (10). Inflammation can increase hepcidin levels, primarily mediated through increased production of interleukin 6 (IL-6), which binds to the gp130 protein receptor complex causing phosphorylation of signal transducer and activation of transcription 3 (STAT3) which then promotes hepcidin transcription (11, 12). Other studies suggest that SIA is independent of drug anti-proliferative effects and that iron homeostasis maybe directly affected by sirolimus as opposed to the development of an inflammatory state (13). A final mechanism proposes the development of erythropoietin (EPO) resistance induced by mTOR inhibitors as a potential cause of SIA (14–16).

The aim of our study was to elucidate the role of mTOR inhibitors on iron, hepcidin and erythropoietin-mediated regulation of hemopoiesis in stable renal transplant recipients (RTR).

## Methods

### Study Design and Patient Selection

We conducted a single-center, prospective case-control study of 26 adult RTR conducted between 2016 and 2019. RTR were on stable mTOR inhibitor (cases, 11/26 [42%]) or tacrolimus dosing (controls, 15/26 [58%]). Patients were recruited opportunistically through routine follow-up clinics following renal transplant. Inclusion criteria for cases were: age >18 years, >1 year post renal transplant on either sirolimus or everolimus, estimated glomerular filtration rate (eGFR) >45 ml/min and no evidence of biopsy proven rejection in the preceding 12 months. Controls received tacrolimus instead of an mTOR inhibitor. Exclusion criteria were: age <18 or >80 years, other causes of anemia such as gastrointestinal bleeding and the presence of active non-skin malignancies. The study was approved by the South Metropolitan Health Service Human Research Ethics Committee and all patients provided written informed consent before participation. Laboratory tests for the study were conducted as part of routine clinical care, except hepcidin, EPO and IL-6 assays which were not routine.

### Data Collection

Baseline demographic data were collected from electronic health records and included age, gender, type of transplant, reason for transplant, years since transplant, immunosuppressive regimen with total daily dose and reason for use of mTOR inhibitor. Co-morbidities and reasons for an active inflammatory state were also recorded. No cases or controls received iron or erythropoietin supplementation in the preceding 12 months.

The preferred maintenance immunosuppression post-renal transplant at our institution is triple therapy with prednisolone, tacrolimus and mycophenolate mofetil (MMF). Non-melanoma skin cancers and early allograft nephropathy are the most common reasons for switching tacrolimus to an mTOR inhibitor. 7/11 (64%) cases were using mTOR inhibitors due to non-melanoma skin cancers, with 2 of these 7 also having a history of additional solid organ malignancies. The remaining 4/11 (36%) were using mTOR inhibitors due to early allograft nephropathy with calcineurin inhibitors. Immunosuppressive regimens were variable for cases; 7/11 (64%) patients were on three agents (prednisolone, MMF and mTOR inhibitor for most), 2/11 (18%) were on two agents (mTOR inhibitor plus prednisolone or MMF) and 2/11 (18%) were on sirolimus monotherapy. Immunosuppressive regimens for controls consisted of three agents for 12/15 (80%) whilst 3/15 (20%) were on prednisolone and tacrolimus only.

Laboratory data included hemoglobin (Hb), mean cell volume (MCV), iron studies (serum iron, ferritin, transferrin and Tsats), creatinine, eGFR, hepcidin-25, EPO and IL-6 levels. eGFR was calculated using the CKD-EPI equation at our institution (17), a reported eGFR >90 ml/min was entered as 90 ml/min for the purpose of data tabulation. All laboratory data for each individual patient were from the same venepuncture.

Serum IL-6 levels were measured by ELISA according to the manufacturer's instructions (Human IL-6 Quantikine HS ELISA kit, R&D Systems, Inc. Minneapolis, USA). The assay had a sensitivity of 0.039 pg/mL (18).

Hepcidin was isolated from serum using a solid phase extraction approach, and measured by liquid chromatography-quadrupole time-of-flight mass spectrometry (LC-qTOF-MS), using a Waters Xevo XS Mass Spectrometer (Waters, Manchester, UK). Quantitation of the hepcidin was by measurement of the accurate mass [M+5H]5+ ion, against a synthetic hepcidin-25 heavy isotope standard (Peptides International, Kentucky, USA) as previously described (19–21).

### Statistical Analysis

Data are presented as mean and median values, with standard deviation (SD) and interquartile range (IQR), as appropriate. Case and control data passed normality testing using Anderson-Darling and D'Agostino-Pearson tests. Groups were compared using unpaired *t*-tests and results were considered significant if the *p*-value was < 0.05. Logistic regression analysis was performed (Prism 9.1, GraphPad Software).

## Results

### Patient Population

There were no differences between cases and controls in terms of age, gender or allograft function (Table 1). Characteristics of renal transplants in each group are described in Table 1. Details of mTOR inhibitor and tacrolimus use, including daily doses and mean trough levels, is summarized in Table 2. Cases were significantly longer post-transplant than controls (9 vs. 3 years; *p* = 0.001).

**Table 1:** Characteristics of RTR subjects included in study.

	**mTOR inhibitor based regimen**				**Tacrolimus based regimen**				***p*-value**
	**Male**		**Female**		**Male**		**Female**		
Gender	6		5		8		7		
Type of transplant	Cadaveric		Live		Cadaveric		Live		
	4		7		8		7		
	Mean	Median	SD	IQR	Mean	Median	SD	IQR	
Age (years)	54	59	15.3	30	49	52	12.4	14	0.37
eGFR (mL/min/1.73 m^2^)	69	62	14.8	34	73	77	18.4	25	0.55
Years post-transplant	9	8	4.9	4	3.5	2	2.7	5	<0.001

**Table 2:** Details of mTOR inhibitor and tacrolimus use.

	**Sirolimus based regimen**	**Everolimus based regimen**	**Tacrolimus based regimen**
No. of subjects	9	2	15
Mean daily dose (mg)	1.72	1	4.3
Mean trough level (ng/mL)	8.46	2.2	5.8

Two cases had underlying inflammatory states; one had chronic *Mycobacterium avium complex* infection on therapy and a second had colitis 1 week prior to venepuncture.

### Red Blood Cell Indices, Iron Studies, Hepcidin, and IL-6

The mean Hb and MCV levels for both cases and controls were within the reference range and there were no significant differences between the two groups (Table 3). There were no significant differences for serum ferritin, transferrin saturation, hepcidin-25 or IL-6 levels between the two groups (Tables 3, 4). The mean EPO levels were significantly higher in the mTOR inhibitor group compared with controls (16.8 vs. 8.49 IU/L, *p* = 0.028). A logistic regression analysis was performed using mTOR use (yes/no), Hb and EPO levels as the variables. EPO level was the only variable that had a significant impact on the model providing an odds ratio of 0.84 (95%CI 0.66–0.98). The area under the receiver operator characteristic curve (ROC) for the analysis was 0.77 (95%CI 0.54–0.94) with *p* = 0.04.

**Table 3:** Red blood cell indices and iron study parameters.

	**mTOR inhibitor based regimen**				**Tacrolimus based regimen**				***p*-value**
	**Mean**	**Median**	**SD**	**IQR**	**Mean**	**Median**	**SD**	**IQR**	
Hb (g/L)	143	142	19.5	30	147	142.5	19.6	21	0.59
MCV (fL)	88	87	6.9	10	90.4	90	5.9	3	0.35
Ferritin (μg/L)	150	116	111	162	85.7	73.5	53.9	90.4	0.06
Transferrin	27.3	29.2	5.6	9.9	31	30.5	5.1	8.4	0.09
Tsats (%)	26	26	7.6	13	23.3	23	9.8	17	0.46

**Table 4:** EPO, hepcidin and IL-6 levels.

	**mTOR inhibitor based regimen**				**Tacrolimus based regimen**				***p*-value**
	**Mean**	**Median**	**SD**	**IQR**	**Mean**	**Median**	**SD**	**IQR**	
EPO (IU/L)	16.8	11.8	13.1	9.6	8.49	8.5	3.94	5.2	0.028
Hepcidin-25 (nM)	3.62	3.20	2.84	2	3.26	2.6	2.89	4.8	0.76
IL-6 (pg/mL)	11	8.5	6.4	7.65	7.02	5.11	6.65	4.7	0.14

## Discussion

Several mechanisms have been suggested to underly the impaired hemopoiesis observed in RTR receiving mTOR inhibitor therapy. These include inflammation, impaired iron or hepcidin metabolism, and dysregulated erythropoietin metabolism. Whilst subjects in our study receiving mTOR inhibitor or tacrolimus therapy had similar hematologic parameters, serum iron biochemistry, renal function, hepcidin and IL-6 levels, we observed that those receiving mTOR inhibitors had higher levels of EPO, suggesting that EPO resistance is induced by mTOR inhibitor treatment. In both groups eGFR was maintained suggesting elevated EPO was not explained by declining renal function. In our study, the elevated EPO production was able to compensate and preserve hemopoiesis. Previous studies have demonstrated that microcytosis or anemia are induced in mTOR inhibitor-treated subjects (7, 13–15, 22–24). EPO is also known to inhibit hepcidin production (25–28). The presence of similar hepcidin levels in the mTOR and tacrolimus groups, despite the elevated EPO levels in the former, suggests that the effects of EPO are probably direct and not mediated via changes in hepcidin production. *In vitro* studies have demonstrated that mTOR inhibitors induce microcytosis, possibly due to inhibition of erythroid precursors (22, 23) and that these effects are not overcome by increasing available iron in the culture environment (23). This microcytosis is reversed by withdrawal of mTOR inhibitors (7, 13, 15).

Chronic inflammation has been implicated in the causation of microcytic anemia secondary to sirolimus. Thanaut et al. observed elevations in ferritin and CRP in patients on sirolimus which fell after withdrawal of the drug (7). A subset analysis in the same study demonstrated elevated IL-6 and TNF∝ measurements that fell with sirolimus withdrawal in six patients. Sirolimus trough levels were higher than those seen in our study. Przybylowski et al. reported elevated hepcidin and IL-6 levels in orthotopic heart transplant patients treated with mTOR inhibitors and suggested that chronic inflammation and increased hepcidin production may be responsible for functional iron deficiency in these patients (29). This study had significant confounding variables, including older age, lower eGFR and higher BNP levels in mTOR inhibitor group. A prospective randomized study by Maiorano et al. produced results consistent with our observations, demonstrating that prohepcidin levels were similar in patients on sirolimus vs. cyclosporin, despite the presence of microcytic anemia in the sirolimus patients, which was not improved with oral iron supplementation (13). However, prohepcidin levels correlate poorly with total body iron status in comparison with mature hepcidin-25 (30). A 2021 pediatric case-control study found no difference in IL-6, hepcidin or ferritin levels in 17 children with tuberous sclerosis complex treated with everolimus compared to 47 controls (23). IL-6 levels were also measured in the patients prior to commencing everolimus and at 3, 6 and 12 months with no significant elevation with introduction of everolimus (23). IL-6 levels were similarly elevated in both our treatment groups, compared to data derived from healthy adults (31), indicating similar levels of inflammation and suggesting this pathway does not contribute to impaired hemopoiesis.

mTOR inhibitors have been shown to inhibit the EPO signaling pathway at the level of EPO receptor signaling (32). In the presence of EPO, the EPO receptor activates multiple phosphorylating enzymes, including phosphoinositide 3-kinase (PI 3-K) which leads to eventual activation of P70S6 kinase with subsequent protein synthesis and cell proliferation (32). mTOR inhibitors are potent inhibitors of P70S6 kinase (33–35). This direct effect on the EPO pathway may explain the previously demonstrated effect of mTOR inhibitors on red cell indices that are disproportionate to their effects on other aspects of bone marrow function. Augustine et al. provided data in support of mTOR inhibitor-induced EPO resistance, demonstrating a fall in EPO resistance when switching 25 stable RTR from sirolimus to MMF (erythropoietin:Hb ratio 2.7 on sirolimus, to 1.2 on MMF (where Hb was measured in g/dL) (14). Our study confirms and extends the observations of Augustine et al., demonstrating elevated EPO levels in the mTOR inhibitor group with similar Hb to the tacrolimus group.

Our study is limited by the small sample size in both our cases and controls and the variable use of sirolimus and everolimus in our cases. The different immunosuppressive regimens used in each group and the two cases of active inflammation in our mTOR inhibitor group are potentially confounding variables that reflect real-world practice with these complex patients. The absence of anemia and microcytosis in this cohort requires these results to be extrapolated to be applied to this cohort as an explanation for this phenomenon. This may have been due to sample size, duration of drug exposure or the target levels achieved being below a threshold for effect. Cases were significantly longer post-transplant than controls which may have influenced results, however, this did not result in a differences in age or allograft function.

In conclusion, higher levels of EPO in the absence of deranged iron biochemistry or hepcidin-25 levels suggest that EPO resistance rather than impaired iron metabolism may contribute to impaired haemopoiesis previously demonstrated in RTR on mTOR therapy.

## Data Availability Statement

The raw data supporting the conclusions of this article will be made available by the authors, without undue reservation.

## Ethics Statement

The studies involving human participants were reviewed and approved by South Metropolitan Health Service Human Research Ethics Committee. The patients/participants provided their written informed consent to participate in this study.

## Author Contributions

HP, JO, AK, AI, and RS designed the study and recruited participants. RJ was responsible for data collection and tabulation and authored the manuscript. JO performed statistics. All authors contributed to interpretation of the data and editing of the manuscript.

## Funding

This project received funding from a Spinnaker Health Research Foundation grant.

## Conflict of Interest

The authors declare that the research was conducted in the absence of any commercial or financial relationships that could be construed as a potential conflict of interest.

## Publisher's Note

All claims expressed in this article are solely those of the authors and do not necessarily represent those of their affiliated organizations, or those of the publisher, the editors and the reviewers. Any product that may be evaluated in this article, or claim that may be made by its manufacturer, is not guaranteed or endorsed by the publisher.
